# Cost-effectiveness of compression technologies for evidence-informed leg ulcer care: results from the Canadian Bandaging Trial

**DOI:** 10.1186/1472-6963-12-346

**Published:** 2012-10-02

**Authors:** Ba' Pham, Margaret B Harrison, Maggie H Chen, Meg E Carley

**Affiliations:** 1 Toronto Health Economics and Technology Assessment Collaborative, Department of Health Policy Management and Evaluation, University of Toronto, Leslie Dan Pharmacy Building, 6th floor, Room 651, 144 College Street, Toronto, ON, M5S 3 M2, Canada; 2 School of Nursing, Queen's University, Kingston, ON, Canada; 3 School of Public Health Sciences, University of Toronto, Toronto, ON, Canada

**Keywords:** Randomized controlled trial, Cost-effectiveness, Leg ulcers, Compression therapy, Community care

## Abstract

**Background:**

Venous leg ulcers, affecting approximately 1% of the population, are costly to manage due to poor healing and high recurrence rates. We evaluated an evidence-informed leg ulcer care protocol with two frequently used high compression systems: ‘four-layer bandage’ (4LB) and ‘short-stretch bandage’ (SSB).

**Methods:**

We conducted a cost-effectiveness analysis using individual patient data from the Canadian Bandaging Trial, a publicly funded, pragmatic, randomized trial evaluating high compression therapy with 4LB (n = 215) and SSB (n = 209) for community care of venous leg ulcers. We estimated costs (in 2009–2010 Canadian dollars) from the societal perspective and used a time horizon corresponding to each trial participant’s first year.

**Results:**

Relative to SSB, 4LB was associated with an average 15 ulcer-free days gained, although the 95% confidence interval [−32, 21 days] crossed zero, indicating no treatment difference; an average health benefit of 0.009 QALYs gained [−0.019, 0.037] and overall, an average cost increase of $420 [$235, $739] (due to twice as many 4LB bandages used); or equivalently, a cost of $46,667 per QALY gained. If decision makers are willing to pay from $50,000 to $100,000 per QALY, the probability of 4LB being more cost effective increased from 51% to 63%.

**Conclusions:**

Our findings differ from the emerging clinical and economic evidence that supports high compression therapy with 4LB, and therefore suggest another perspective on high compression practice, namely when delivered by trained registered nurses using an evidence-informed protocol, both 4LB and SSB systems offer comparable effectiveness and value for money.

**Trial registration:**

ClinicalTrials.gov Identifier: NCT00202267

## Background

Community care of individuals with chronic wounds is an important issue for community care authorities as it consumes approximately 14% of their budget [1]. In particular, budget shortfalls and nursing shortages make caring for individuals with venous leg ulcers a challenge [2]. This chronic condition affects approximately 1% of people at some time in their lives [3]. It is associated with poor healing [4], high recurrence [5], and negative impact on physical and psychological wellbeing [6].

Best practice supported by high level evidence recommends high compression bandaging applied by well-trained clinicians for individuals with leg ulcers associated with venous insufficiency [7-11]. Compression bandaging systems are all designed to improve venous return but vary by construction (knitted, woven), components (elastic, non-elastic), performance (long-stretch, short-stretch) and layers (single-layer, multiple-layer) [12]. Some frequently used systems are the “four-layer bandage” system (strictly speaking a four-component system, 4LB) and the multi-component compression that includes a “short-stretch bandage” (SSB) [12]. The Canadian Bandaging Trial (CBT, n = 424 participants), funded by the Canadian Institutes of Health Research, was a pragmatic, multi-centre (10 centres in three provinces), open-label, randomized comparative trial evaluating the effectiveness and cost-effectiveness of high compression therapy with 4LB and SSB for community care of venous leg ulcers [13,14]. Results of the effectiveness analysis have been recently published [13]. On average, leg ulcers in participants treated with 4LB heal slightly faster than those in SSB participants but overall, the treatment groups are not significantly different with respect to healing times, recurrence rates, health-related quality of life, or pain [13]. We described results of the cost-effectiveness analysis below.

## Methods

### Overview

This cost-effectiveness analysis compared the costs and quality-adjusted life years (QALYs) of high compression therapy with 4LB and SSB for individuals with venous leg ulcers using individual patient data from the CBT. The comparison was conducted within the following decision-making context: community care setting (home or nursing clinic); participating centres were supported during the pilot phase of the trial to develop a common evidence-informed protocol for venous leg ulcers (e.g., full assessment, ankle brachial pressure index to screen for arterial disease) [14]; and registered nurses (RNs) with the trial were trained on the evidence-informed protocol to reduce variation in service delivery and bandaging skills [13,14]. Ethics approval for the trial was received from Queen’s University Research Ethics Board, Kingston Canada (REB# NURS-140-03).

We conducted the analysis according to the intention-to-treat principle [15]. Given the funding source, we estimated costs from the societal perspective in the base case analysis and given the decision-making context, considered the health systems and community care perspectives in scenario analyses [16]. All costs are expressed in 2009–2010 Canadian dollars. We measured QALYs during the first year of the trial and therefore did not use discounting of future outcomes [16].

### Patient population

The patient population is described elsewhere [13]. In total, 424 consenting individuals referred for community care with a venous leg ulcer were randomized to 4LB (n = 215) and SSB (n = 209). In total, 23 (11%) 4LB participants were lost to follow-up and 57 (27%) participants discontinued therapy before healing. The corresponding number for SSB participants was 10 (5%) and 44 (21%). Reasons for lost to follow-up and discontinuation are reported elsewhere [13]. Participants were on average 65 years of age, of whom 80% were fully mobile.

### High compression therapy

In the late 1990’s, best practice recommendations in Canada and elsewhere, supported by high level evidence, indicate that the most effective treatment for venous leg ulcers is high compression bandaging [7,17-19] applied by well-trained healthcare professionals [20]. By 2004 when the CBT trial started, high compression bandaging had become the standard of care, although it was unclear which high compression systems should be used in routine practice [21].

In the CBT, community care RNs trained in both the 4LB and SSB systems administered the randomly allocated high compression system to participants. The 4LB system is available as a kit (i.e., Profore®, Smith & Nephew Medical Ltd.) and is used once and discarded. There is no pre-packaged SSB system. Instead, the attending nurses selected the bandages (e.g., the initial protective padding layer covered with two bandaging layers) depending on a participant’s leg circumferences. Whenever possible, SSB bandages (e.g., Comprilan®, Beiersdorf-Jobst, Inc.) were washed by the participants and reused.

### Resources used

Resources utilization data were collected from baseline to healing or 12 months whichever came first. In the CBT, visit schedule (at least once a week) was not dictated by the trial protocol but rather determined by the visiting nurse. At each visit, nursing staff completed a clinical sheet detailing the care given (e.g. dressings, skin treatments), the approximate visit time, and also documented on a supply log all newly opened supplies provided by the community care authority (e.g., compression bandages, wound contact layers, cohesive bandages).

Monthly and at the time of healing, participants filled out expense forms for resources consumed or expenses incurred *because* of leg ulcers, including health services used (i.e., visits to hospitals, family physicians, specialists, and emergency rooms), out-of-pocket expenses (i.e., taxi fares, parking fees, and preventive supplies (e.g., stocking, alternate shoes)), hire help (e.g., cleaning, meal preparation, gardening, and snow shovelling) and lost work days.

We assumed that other resources unrelated to leg ulcers had been unchanged with treatment allocation.

### Unit costs

Table 1 displays unit costs of the resources used for leg ulcer care. The site-specific unit costs of treatment supplies were obtained from the participant centres. The average cost of the 4LB kit was approximately $30 (range: $23, $41). The cost of SSB depends on how the system is assembled for a participant’s leg circumferences (e.g., 6- and 8-centimeter bandages and padding). Its cost was participant-dependent, ranging on average from $29 to $35 (overall range: $20, $42).

**Table 1:** **Unit prices used to value resources consumed (2009–2010 Canadian dollar)**

**Item of resource**	**Unit**	**Unit cost ($)**	**Source**
High compression bandages			
Four-layer bandage (4LB) system	Kit	29.55	Site data^*^
Short-stretch bandage (SSB) system			
6 cm bandage	Each	11.69	Site data^*^
8 cm bandage	Each	13.90	Site data^*^
10 cm bandage	Each	14.89	Site data^*^
12 cm bandage	Each	17.27	Site data^*^
Padding	Each	2.91	Site data^*^
Typically used SSB system			
6 cm + 8 cm + padding	System	28.50	Site data^*^
8 cm + 10 cm + padding	System	31.70	Site data^*^
10 cm + 12 cm + padding	System	35.07	Site data^*^
Hourly nursing wage	Hour	35.15	ONA^†^
Health services utilization			
Family doctor visit	Visit	34.70	OHIP^‡^
Specialist visit	Visit	68.31	OHIP^‡^
Emergency room visit	Visit	252.00	OHIP^‡^
Outpatient hospital visit	Visit	426.00	OCCI^§^
Time cost of lost work due to leg ulcer	Hour	9.41	HRSD^¶^

*Abbreviations:* ONA: Ontario Nursing Association. OCCI: Ontario Case Costing Initiative. OHIP: Ontario Health Insurance Plan database. HRSD: Department of Human Resources and Skills Development. Notes:^*^Unit prices are for illustration purposes only; the analysis uses site-specific prices (see Methods). ^†^Average full-time hourly rate of an RN [22]. ^‡^Mean costs of physician services from OHIP database (see Methods). ^§^Average direct (and overhead) cost of ambulatory care visits [25]. ^¶^Average minimum hourly wages across provinces [26].

The average hourly wage of an RN was $35 ($30, $42) [22]. Average unit costs for physician services (family physicians, specialists, and emergency departments - EDs) were obtained from the Ontario Health Insurance Plan dataset [23]. A location code in the dataset indicates whether the service was provided in a physician’s office or in an ED. Costs reflect mean fee paid per visit (e.g., $52 for physician cost per ED visit). The total average cost per ED visit includes an additional $200 for non-physician costs [24]. The mean direct cost (including overhead costs) of outpatient hospital (ambulatory care) visits was obtained from the Ontario Case Costing Initiative [25]. The time cost of lost work was valued using the average minimum wage [26].

### Cost estimates

From the community care perspective, the costs of nursing visits and all treatment supplies provided by the community care authorities were included. The health system costs included the community care costs and visit costs to outpatient services, family physicians, specialists and emergency rooms. The societal costs included the health system costs and all expenses and lost income related to leg ulcers incurred by the participants.

### Quality-adjusted life-years

QALYs were derived for all participants to reflect survival time, treatment outcomes and health-related quality of life according to the EQ-5D™ questionnaire [27]. The EQ-5D™ is a generic measure of health status, where health is characterised on five dimensions (mobility, self care, ability to undertake usual activities, pain, anxiety / depression) [28]. Each dimension has 3 levels, reflecting “no health problems,” “moderate health problems,” and “extreme health problems.” Each response placed a participant into one of 243 mutually exclusive health states, each of which has previously been valued on the 0 (equivalent to dead) to 1 (equivalent to good health) ‘utility’ scale to derive a health-related quality-of-life weight from a sample of 4048 members of the US public [29]. We used the US valuation scheme because at the time of the analysis a Canadian scheme is still under-development (Dr. Jeffrey A. Johnson, University of Alberta, personal communication, April 3, 2010).

Participants filled out the EQ-5D™ questionnaire at baseline, every three months while on treatment or at healing time and 3 months post-healing. Over one year, each participant had a quality-of-life weight from three to five time-points and, by using area under the curve methods which effectively weights time by health-related quality of life, we derived QALYs [30]. To account for baseline variation in quality-of-life weights, we estimated mean QALYs for treatment groups adjusting for baseline differences [31].

### Data analysis

Scenario analyses were conducted by varying perspectives to inform different decision makers. We presented the results separately for societal, health system and community care perspectives. We conducted the base case analysis using site-specific wholesale prices of all treatment supplies, accounting for variation in the unit prices of 4LB kits, and variation in the types of SSB bandages assembled according to individual participants’ leg circumferences and their varied unit prices. Sensitivity analyses were conducted 1) using average, low and high prices of the 4LB kit and 2) using highest unit prices (of the site-specific prices) of the bandages and padding assembled for the SSB systems.

Relative to SSB, the average ulcer-free days gained with 4LB was estimated by the median difference in the time to healing using the Kaplan-Meier method [15]. The mean costs and mean QALYs of 4LB and SSB were estimated using methods to adjust for censored data due to lost to follow-up [32-34]. Patients who discontinued their allocated bandages continued to be followed; their data were included in the analysis, conducted according to the intention-to-treat principle [15]. We estimated the differential mean costs and mean QALYs of 4LB relative to SSB, and derived the corresponding incremental cost effectiveness ratios (ICERs).

To account for sampling variation, we re-sampled the individual participant data from the original CBT with replacement to create 1000 bootstrapped CBT trials and re-analyzed the bootstrapped trial data [35,36]. For simplicity, we did not take into account variation in the unit costs of health services used and hourly wages (Table 1). We derived the 95% “credible” (hereafter “confidence”) intervals for differential costs and QALYs using the bias corrected and accelerated bootstrap method [35]. We plotted cost effectiveness acceptability curves [37,38], showing the probability that 4LB is more cost effective than SSB for different values decision makers are willing to pay for an additional QALY gained with compression therapy.

All analyses were conducted in R, version 2.9.0, R Foundation for Statistical Computing.

## Results

### Resources used

Table 2 summarizes resources used and time loss *due* to leg ulcers. Relative to SSB, 4LB was associated with a 14% decrease in the average number of nursing visits and average total nursing time. On average, participants used approximately twice as many 4LB as SSB bandages. 4LB participants visited outpatient services, family physicians, specialists and emergency rooms less often than SSB participants, although the annual utilization rates were very small. There were no reported hospitalizations and the mean numbers of lost work hours were very small.

**Table 2:** **Resources used and time loss due to leg ulcers during the first year of follow-up**

	**4LB (n = 215)**	**SSB (n = 209)**	Difference^*^
	**Mean (SD)**	**Mean (SD)**	
Number of nursing visits	28.25 (24.16)	32.64 (24.07)	−14%
Total nursing time (hour)	15.90 (13.28)	18.48 (15.63)	−14%
Total number of bandages used	24.04 (24.54)	11.64 (10.07)	107%
Health services used			
Family doctor visits^†^	0.284 (0.790)	0.368 (0.857)	−23%
Specialist visits^†^	0.019 (0.135)	0.081 (0.402)	−77%
Hospital visits^†^	0.005 (0.068)	0.014 (0.119)	−64%
Emergency room visits^†^	0.260 (0.087)	0.411 (1.603)	−37%
Lost work hours^‡^	1.14 (6.09)	1.24 (5.29)	−8%

*Abbreviations:* 4LB: four-layer bandage. SSB: short-stretch bandage. SD: standard deviation. Notes:^*^(4LB mean - SSB mean), expressed as percentages of the SSB mean. ^†^Total number of visits per participant. ^‡^Total number of hours missed work due to leg ulcer per participant.

### Costs

Table 3 shows mean costs per participant. The marked differences were in high compression bandaging cost ($606 more with 4LB, because twice as many 4LB bandages were used), nursing time cost ($90 less with 4LB, mainly due to the average ulcer-free days gained with 4LB, as reported below), and out-of-pocket expenses ($30 less with 4LB). Overall, 4LB cost more per participant per year (point estimate: $420; 95% confidence interval: $235 to $739).

**Table 3:** **Mean costs during the first year of follow-up**

	**4LB (n = 215)**	**SSB (n = 209)**	Cost Difference $^*^
	**Mean cost (SD) $**	**Mean cost (SD) $**	
Nursing visits	563 (463)	653 (547)	−90
Compression bandages	811 (897)	205 (232)	606
Health services used^†^	35 (96)	46 (117)	−11
Out-of-pocket expenses^‡^	28 (78)	58 (177)	−30
Lost work due to leg ulcers	1.82 (8.08)	2.39 (9.06)	−0.57
Hired help^§^	0.43 (3.25)	0.56 (2.98)	−0.13
	Mean (95% CI)	Mean (95% CI)	
Total cost	1570 (1448, 1870)	1150 (1014, 1297)	
Differential cost	420 (235, 739 )		

*Abbreviations:* 4LB: four-layer bandage. SSB: short-stretch bandage. SD: standard deviation. CI: confidence intervals derived from the 1000 bootstrapped trials. Notes:^*^(4LB mean – SSB mean). ^†^Per participant costs of visits to family physicians, specialists, outpatient services, and emergency rooms. ^‡^Per participant expenses related to leg ulcer care. ^§^Costs of hired help related to leg ulcer.

In summary, bandaging costs were substantially higher for 4LB and these were only partially offset by the lower health utilisation costs for 4LB compared with SSB.

### Health outcomes

Table 4 summarizes healing time and QALYs. Leg ulcers treated with 4LB on average healed slightly faster than those with SSB, resulting in an average 15 ulcer-free days gained with 4LB; however, the 95% confidence interval (−32 days, 21 days) crossed zero, indicating no statistically significant difference between treatment groups. Healing rates were also not different between treatments: at 12 weeks, the healing rate was 58% for 4LB and 53% for SSB; the corresponding rate was 71% and 78% at 26 weeks and 83% and 92% at 52 weeks (data not shown).

**Table 4:** **Summary of healing time and QALYs during the first year of follow-up**

	**4LB (n = 215)**	**SSB (n = 209)**
	**Median (95% CI)**	**Median (95% CI)**
Healing time (days)	62 (51, 73)	77 (63, 91)
Ulcer-free days	−15 (−32, 21)	
	Mean (95% CI)	Mean (95% CI)
Quality-adjusted life years	0.839 (0.811, 0.862)	0.830 (0.809, 0.851)
Difference (QALYs)	0.009 (−0.019, 0.037 )	

*Abbreviations:* 4LB: four-layer bandage. SSB: short-stretch bandage. CI: confidence interval derived from the 1000 bootstrapped samples. QALY: Quality-adjusted life year.

**Figure 1:**
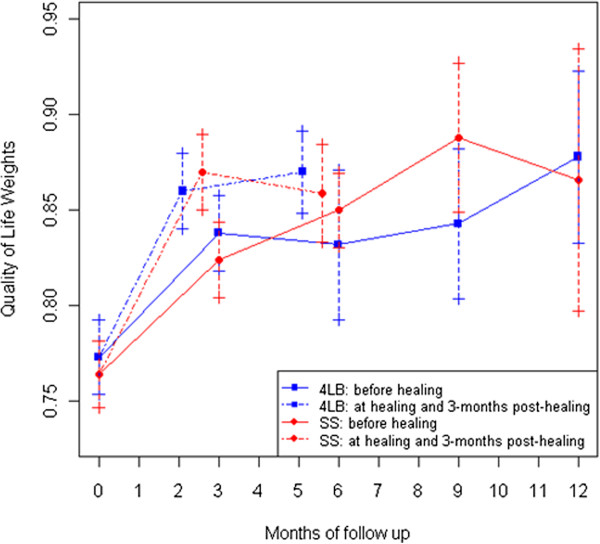
Mean quality of life weights^*^ by treatment group during the first year of follow-up.*Abbreviations:* 4LB: four-layer bandage. SSB: short-stretch bandage. *Notes:* Error bars denote standard error of the means. ^*^Health-related quality of life according to the EQ-5D™ questionnaire [27].

The mean quality-of-life weights were comparable for both groups at regular assessment intervals while participants were on treatment (Figure 1, solid lines). Both groups attained similar improvement in quality-of-life weights at healing and sustained the improvement comparably at 3 months post healing (Figure 1, broken lines), partially reflecting similar recurrence rates (10% for 4LB and 13% for SSB at 52 weeks, data not shown). Comparable trends in health-related quality of life between groups were also observed with the physical and mental components of the SF-12 (data not shown).

Partially due to the average 15 ulcer-free days gained with 4LB, 4LB was associated with a differential mean of 0.009 QALYs (95% confidence interval: -0.019, 0.037 QALYs, Table 4), corresponding to 3.3 quality-adjusted life-days gained (−7.0, 13.5).

### Cost effectiveness

For the base case analysis, both mean overall costs and mean QALYs were higher for 4LB than for SSB participants, with mean differences of $420 and 0.009 QALYs. Here, the issue is whether decision makers are willing to pay $420 more for the 0.009 QALYs gained, or equivalently, the additional cost of $46,667 ($420/0.009) per QALY gained associated with 4LB — the implied ICER.

Using a willingness-to-pay value of $50,000 per QALY as an exchange rate, the health benefit of 0.009 QALYs gained is equivalent to $450 and the net monetary benefit associated with 4LB was $30 ($450 - $420). Alternatively, the additional cost of $420 is equivalent to 3.1 quality-adjusted life days and the net health benefit was 0.2 (approximately 3.3 – 3.1) quality-adjusted life days. At a willingness-to-pay value of $100,000 per QALY, the corresponding net monetary benefit was $480 and the corresponding net health benefit was 1.8 quality-adjusted life days.

**Figure 2:**
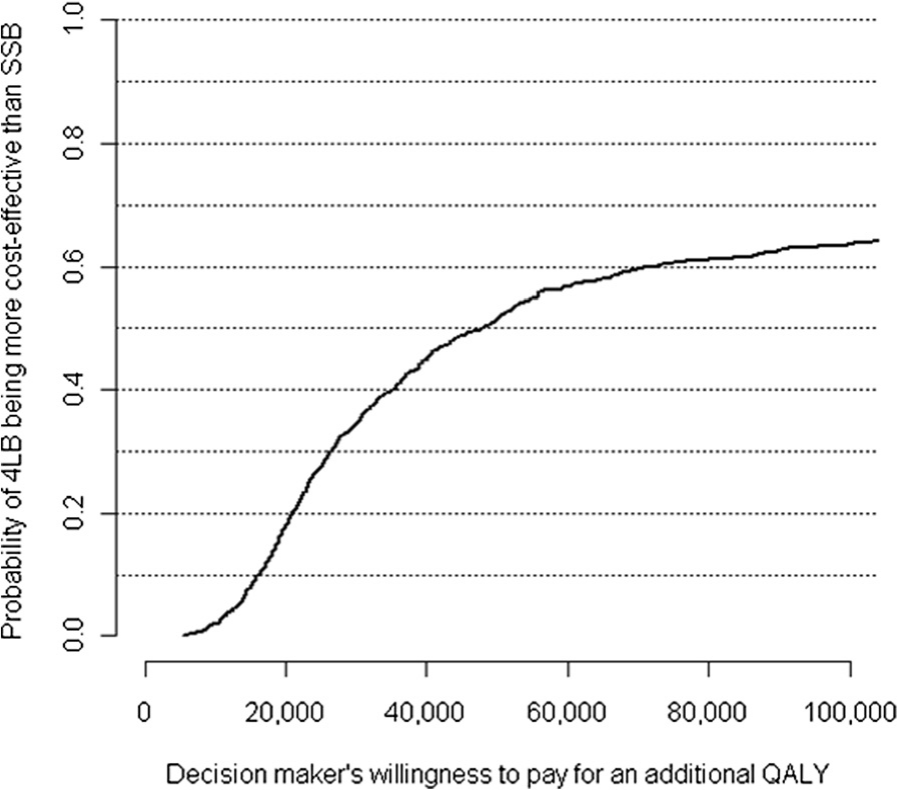
**Probability of 4LB being more cost-effective at different willingness-to-pay values.***Abbreviations:* 4LB: four-layer bandage. SSB: short-stretch bandage.

Above, the ICER was estimated with sampling uncertainty, which is represented in Figure 2 in the form of a cost effectiveness acceptability curve. The curve indicates that the higher the value decision makers are willing to pay for an additional QALY gained, the higher the probability that 4LB will be more cost effective than SSB. For willingness-to-pay values ranging from $50,000 to $100,000 per QALY gained, the probability that 4LB is more cost effective than SSB increased from 51% to 63%.

### Scenario and sensitivity analyses

Table 5 displays results of the scenario and sensitivity analyses. The ICER was approximately $48,000 from a health system perspective and approximately $51,000 from a community care perspective. When the unit price of the 4LB kit was fixed at its average ($30), lowest ($23) and highest ($39) price, the ICERs changed slightly from approximately $46,000 to $47,000. Using the highest unit prices of the components of the SSB systems (instead of the site-specific unit prices of the components), the ICER was approximately $45,000.

**Table 5:** Results of base case, scenario and sensitivity analyses^*^

	**4LB (n = 215)**	**SSB (n = 209)**	**Differential cost**	**Cost per QALY gained**
	**Mean cost (95% CI)**	**Mean cost (95% CI)**	**Mean (95% CI)**	
Base case analysis^‡^	1570 (1448, 1870)	1150 (1014, 1297)	420 (235, 739 )	46,667
Scenario analyses				
Community care perspective	1497 (1371, 1760)	1034 (930, 1169)	463 (308, 748)	51,444
Healthcare payer perspective	1519 (1394, 1786)	1089 (968, 1216)	430 (2801, 749)	47,777
Sensitivity analyses				
Unit price of a 4LB kit				
Average ($29.55)^†^	1567 (1442,1879)	1150 (1014, 1297 )	417 (256, 753)	46,333
Low ($22.70)^†^	1561 (1433, 1849 )	1150 (1014, 1297 )	410 (251, 742)	45,556
High ($39.10)^†^	1576 (1459,1864)	1150 (1014, 1297 )	426 (246, 754)	47,333
Highest prices of SSB systems	1570 (1448, 1870)	1169 (1035, 1315)	401 (240, 729)	44,556

*Abbreviations:* 4LB: four-layer bandage. SSB: short-stretch bandage. CI: confidence interval. Notes:^*^Only mean costs are displayed in Table 5; refer to Table 4 for mean QALYs. ^‡^According to the societal perspective. ^†^Average costs of the 4LB kit from site-specific prices.

## Discussion

We evaluated the cost-effectiveness of high compression therapy for community care of individuals with venous leg ulcers. The effects of the two high compression systems were similar. Relative to the SSB system, the 4LB system was associated with a very small health benefit (approximately 3 quality-adjusted life-days gained over one year), and with a small cost increase (an average increase of $420 per individual per year), or equivalently, a cost of approximately $47,000 per QALY gained. For willingness-to-pay values between $50,000 and $100,000 per QALY, the probability that 4LB is more cost effective than SSB ranged from 51% to 63%, indicating that the decision about value for money is finely balanced. Our results are different from recently published studies and therefore suggest another perspective on high compression practice.

Existing clinical and economic evidence supports high compression therapy with 4LB for venous leg ulcers. Results from a meta-analysis including individual patient data from 90% of known randomized patients (or 5 trials) show that relative to SSB, 4LB is associated with significantly shorter healing time of approximately 30% and the treatment effect is consistent across patients with different prognostic profiles [39]. Similar results are also reported in a related Cochrane systematic review (6 randomized trials, a total of 847 participants), but care setting remains an important factor for variation in clinical outcomes [40]. A cost-effectiveness analysis using individual patient data from the largest trial in the review [41], the VenUS I randomized trial (n = 387 participants) also shows that 4LB is more effective and less costly than SSB for community care of venous leg ulcers in the UK [42]. On the other hand, results from the CBT (424 participants) show that in the practice context of trained RNs using an evidence-informed protocol, the choice of high compression bandage system does not materially affect healing times, recurrence rates, health-related quality of life, or pain [13]. Using CBT data, our results show that SSB is as economically attractive as 4LB. Within the right organization of care, the choice of high compression bandaging systems appears to be less important. If delivery of only one, or the other, is possible because of local factors and resources, the CBT results suggest that the expected health benefits and costs would not be compromised whichever is selected.

O’Meara et al. conducted a systematic review (and meta-analysis of randomised controlled trials with data from individual patients) evaluating 4LB and SSB for venous leg ulcers [39]. They identified seven eligible trials (887 patients, and patient level data were retrieved for 5 trials, including 797 patients) and reported that 4LB was associated with significantly shorter time to healing: adjusted hazard ratio 1.31 (95% confidence interval: 1.09 to 1.58). The CBT increases the information available by approximately 50%. Adding the CBT data to the existing evidence in a meta-analysis conducted by CBT members (Drs. Andrea Nelson and Margaret Harrison, Journal of Community Nursing, manuscript submitted), the hazard ratio estimate is 1.14 (0.98, 1.32). The updated evidence suggests that high compression is the key to venous leg ulcer care and the method of delivery is less important than indicated in some earlier work [39-42].

### Limitations

There are a number of important limitations. First, the CBT was open-labelled. Blinding the nurse to the compression therapy was not feasible and once bandage were applied it would have been excessively intrusive to remove them solely for the purpose of an outcome assessment [13]. Healing assessment however was validated by photos assessed by an expert who was located remotely from the site of care and blinded to treatment allocation [13]. We expected minimal bias potentially introduced by the open-label manoeuvre since i) the primary analysis was based upon time to healing and ii) the choice of bandage system did not seem to materially affect healing times, recurrence rates, health-related quality of life, or pain [13].

In mid 2011, we analyzed our EQ-5D data using preference values of EQ-5D health states derived for the US public [29]. In February 2012, the preference valuation for the Canadian public became available [43]. According to Bansback et al. 2012, the correlation between the US and Canadian tariffs is high (i.e., 0.96), although the maximum absolute difference could be large (i.e., 0.06). This is primarily because i) compared to US participants, Canadian participants revealed lower preference values for severe health states and ii) different transformations were applied to values of worse-than-dead states (a linear and monotonic transformation was used for the US and Canadian valuation, respectively so that these values are bounded between −1 and 0). While these are important measurement issues, they would be far less important for the average results in our cost-effectiveness analysis. This was partially because CBT participants 1) were relatively young (i.e., 65 years of age, relative to for example, approximately 71 for a similar trial, the VenUS I trial), 2) were relatively mobile (i.e., 80% relative to 61% for VenUS I), and 3) were with relatively high average EQ-5D valuation at baseline (i.e., 0.77 versus 0.60 for VenUS I) [41,42]. In addition, because the health-related quality of life profiles (i.e., EQ-5D, SF-12 mental and physical component scores) were not markedly different between treatment groups, we did not anticipate any major impacts of this limitation on the results of our analysis.

We valued collected resources that were deemed attributable to leg ulcers, according to the participants. We did not assess the reliability of this attribution although according to our collective experience with conducting the VenUS I and CBT trials, participants would recall most high-value items associated with their leg ulcers.

Last but not least, we only evaluated two frequently used high compression systems (4LB and SSB) although there are a large number of systems available, including newer systems [12].

## Conclusions

Our findings differ from the emerging clinical and economic evidence that supports high compression therapy with 4LB, and therefore suggest another perspective on high compression practice. Namely when delivered by trained registered nurses using an evidence-informed protocol, both 4LB and SSB bandages offer comparable effectiveness and value for money.

## Competing interests

All authors wish to declare that to the best of our knowledge, no conflict of interest, financial or other, exists.

## Authors’ contributions

BP developed the analysis plan for the cost-effectiveness analysis, coordinated the project, wrote the manuscript, and was responsible for the cost-effectiveness analysis and interpretation of the results. MBH was the principal investigator of the Canadian Bandaging Trial (CBT) and responsible for the conceptualization, ethical approval, conduct and management of the CBT. She contributed to the conceptualization of the cost-effectiveness analysis and interpretation of the results. MHC conducted the cost-effectiveness analysis, and assisted with the interpretation of the results. MEC was responsible for data management of the CBT, contributed to the conceptualization of the cost-effectiveness analysis, assisted with the analysis, and contributed to the interpretation of the results. The ‘Canadian Bandaging Trial Group’ members served as site investigators, contributed to the feasibility and conduct of the trial protocol, and contributed to the interpretation of the results of the cost-effectiveness analysis. All primary authors participated in the revisions of the manuscript and approved the final manuscript.

## Pre-publication history

The pre-publication history for this paper can be accessed here:

http://www.biomedcentral.com/1472-6963/12/346/prepub

## References

[B1] Ontario Association of Community Care Access Centres Integrated client care project: from theory to practice 2009 http://www.ccac-ont.ca/Upload/on/General/Integrated_Client_Care_Project_Resource_Table_Presentation_09aug6.pdf. 12-1-2011

[B2] Shannon RJ A cost utility evaluation of best practice implementation of leg and foot ulcer care in the ontario community Wound Care Can 2007 5 S53 S56

[B3] Ruckley CV Socioeconomic impact of chronic venous insufficiency and leg ulcers Angiology 1997 48 67 69 10.1177/000331979704800111 8995346

[B4] Lambourne LA Moffatt CJ Jones AC Dorman MC Franks PJ Clinical audit and effective change in leg ulcer services J Wound Care 1996 5 348 351 8954424

[B5] Callam MJ Harper DR Dale JJ Ruckley CV Chronic ulcer of the leg: clinical history Br Med J 1987 294 1389 1391 10.1136/bmj.294.6584.1389 3109669 PMC1246555

[B6] Persoon A Heinen MM van der Vleuten CJ de Rooij MJ van de Kerkhof PC van Achterberg T Leg ulcers: a review of their impact on daily life J Clin Nurs 2004 13 341 354 10.1046/j.1365-2702.2003.00859.x 15009337

[B7] Fletcher A Cullum N Sheldon TA A systematic review of compression treatment for venous leg ulcers BMJ 1997 315 576 580 10.1136/bmj.315.7108.576 9302954 PMC2127398

[B8] O'Brien JF Grace PA Perry IJ Hannigan A Clarke MM Burke PE Randomized clinical trial and economic analysis of four-layer compression bandaging for venous ulcers Br J Surg 2003 90 794 798 10.1002/bjs.4167 12854102

[B9] Registered Nurses Association of Ontario (RNAO) Nursing best practice guideline: assessment and management of venous Leg ulcers 2004 Toronto, Ontario: Registered Nurses Association of Ontario

[B10] National Health Services Centre for Reviews and Dissemination Compression therapy for venous Leg ulcers Effective Health Care Bulletin 1997 3 1 12

[B11] Royal College of Nursing (RCN) Institute Clinical practice guideline. The management of patients with venous leg ulcers. Recommendations for assessment, compression therapy, cleansing, debridement, dressing, contact sensitivity, training/education and quality assurance 1998 London, England: RCN Institute

[B12] Sibbald R Alavi A Norton L Brown A Coutts P Krasner D, Rodeheaver G, Sibbald RG, Malvern PA Compression therapy Chronic wound care: a clinical source book for healthcare professionals 2007 4 HMP Communications 481 488

[B13] Harrison MB VanDenKerkhof EG Hopman WM Graham ID Carley ME Nelson EA the Canadian Bandaging Trial Group (CBTG) The canadian bandaging trial: evidence-informed leg ulcer care and the effectiveness of two compression technologies BMC Nurs 2011 10 20 10.1186/1472-6955-10-20 21995267 PMC3214126

[B14] Smith LJ Harrison MB Graham ID Lamb M Community leg ulcer bandaging study: lessons learned in a pilot, randomized controlled trial Ostomy Wound Manage 2010 56 32 42 20855910

[B15] Willan A Briggs A Statistical analysis of cost-effectiveness data 2006 New York: John Wiley

[B16] Drummond M O'Brien B Stoddart G Torrance G Methods for the economic evaluation of health care programmes 1997 Oxford: Oxford University Press

[B17] Clinical Resource Efficiency Support Team (CREST) Guidelines for the assessment and management of leg ulceration. Recommendations for practice 1998 Available from URL: http://www.n-i.nhs.uk/crest

[B18] McInnes E Cullum N Nelson A Duff L RCN guideline on the management of leg ulcers Nurs Stand 1998 13 61 63 9923348 10.7748/ns1998.11.13.9.61.c2563

[B19] Kunimoto B Cooling M Gulliver W Houghton P Orsted H Sibbald RG Best practices for the prevention and treatment of venous leg ulcers Ostomy Wound Manage 2001 47 34 50 11235498

[B20] Nelson EA Ruckley CV Barbenel JC Improvements in bandaging technique following training J Wound Care 1995 4 181 184 7600358 10.12968/jowc.1995.4.4.181

[B21] Cullum N Nelson EA Fletcher AW Sheldon TA Compression for venous leg ulcers Cochrane Database Syst Rev 2001 2 CD000265 11405957 10.1002/14651858.CD000265

[B22] Ontario Nurses' Association Frequently asked questions, salary 2010 http://www.ona.org/faqs.html#f16. 17-9-2010

[B23] Ontario Ministry of Health and Long-Term Care Ontario health planning data guide: ministry of health and long-term care ontario 2006 Toronto, Ontario: Ontario Ministry of Health and Long-Term Care

[B24] Ontario Joint Policy and Planning Committee JPPC rate model results based on 2006/07 data: ontario joint policy and planning committee 2008 Toronto, Ontario: JPPC Accountability Committee

[B25] Ontario Case Costing Initiative OCCI costing analysis tool 2011 http://www.occp.com/mainPage.htm. 12-11-2010

[B26] Human R Skills Development C Hourly minimum wages in CANADA for adult workers, 2005–2014 2011 http://srv116.services.gc.ca/dimt-wid/sm-mw/rpt2.aspx?lang=eng&dec=5. 17-9-2010

[B27] Drummond M Introducing economic and quality of life measurements into clinical studies Ann Med 2001 33 344 349 10.3109/07853890109002088 11491193

[B28] Williams A EuroQol–a new facility for the measurement of health-related quality of life The EuroQol Group. Health Policy 1990 16 199 208 10.1016/0168-8510(90)90421-9 10109801

[B29] Shaw JW Johnson JA Coons SJ US valuation of the EQ-5D health states: development and testing of the D1 valuation model Med Care 2005 43 203 220 10.1097/00005650-200503000-00003 15725977

[B30] Matthews JN Altman DG Campbell MJ Royston P Analysis of serial measurements in medical research BMJ 1990 300 230 235 10.1136/bmj.300.6719.230 2106931 PMC1662068

[B31] Manca A Hawkins N Sculpher MJ Estimating mean QALYs in trial-based cost-effectiveness analysis: the importance of controlling for baseline utility Health Econ 2005 14 487 496 10.1002/hec.944 15497198

[B32] Willan AR Lin DY Manca A Regression methods for cost-effectiveness analysis with censored data Stat Med 2005 24 131 145 10.1002/sim.1794 15515137

[B33] Willan AR Lin DY Cook RJ Chen EB Using inverse-weighting in cost-effectiveness analysis with censored data Stat Methods Med Res 2002 11 539 551 10.1191/0962280202sm308ra 12516988

[B34] Lin DY Feuer EJ Etzioni R Wax Y Estimating medical costs from incomplete follow-up data Biometrics 1997 53 419 434 10.2307/2533947 9192444

[B35] Barber JA Thompson SG Analysis of cost data in randomized trials: an application of the non-parametric bootstrap Stat Med 2000 19 3219 3236 10.1002/1097-0258(20001215)19:23<3219::AID-SIM623>3.0.CO;2-P 11113956

[B36] Barber JA Thompson SG Analysis and interpretation of cost data in randomised controlled trials: review of published studies BMJ 1998 317 1195 1200 10.1136/bmj.317.7167.1195 9794854 PMC28702

[B37] Fenwick E Byford S A guide to cost-effectiveness acceptability curves Br J Psychiatry 2005 187 106 108 10.1192/bjp.187.2.106 16055820

[B38] Fenwick E Claxton K Sculpher M Representing uncertainty: the role of cost-effectiveness acceptability curves Health Econ 2001 10 779 787 10.1002/hec.635 11747057

[B39] O'Meara S Tierney J Cullum N Bland JM Franks PJ Mole T Scriven M Four layer bandage compared with short stretch bandage for venous leg ulcers: systematic review and meta-analysis of randomised controlled trials with data from individual patients BMJ 2009 338 b1344 10.1136/bmj.b1344 19376798 PMC2670366

[B40] O'Meara S Cullum NA Nelson EA Compression for venous leg ulcers Cochrane Database Syst Rev 2009 1 CD000265 19160178 10.1002/14651858.CD000265.pub2

[B41] Nelson EA Iglesias CP Cullum N Torgerson DJ Randomized clinical trial of four-layer and short-stretch compression bandages for venous leg ulcers (VenUS I) Br J Surg 2004 91 1292 1299 10.1002/bjs.4754 15382102

[B42] Iglesias CP Nelson EA Cullum N Torgerson DJ Economic analysis of VenUS I, a randomized trial of two bandages for treating venous leg ulcers Br J Surg 2004 91 1300 1306 10.1002/bjs.4755 15382101

[B43] Bansback N Tsuchiya A Brazier J Anis A Canadian valuation of EQ-5D health states: preliminary value set and considerations for future valuation studies PLoS One 2012 7 e31115 10.1371/journal.pone.0031115 22328929 PMC3273479

